# Localized osmotic stress activates systemic responses to N limitation in *Medicago truncatula–Sinorhizobium* symbiotic plants

**DOI:** 10.3389/fpls.2023.1288070

**Published:** 2023-11-20

**Authors:** Marie-Laure Martin, Marjorie Pervent, Ilana Lambert, Stefano Colella, Mathilde Tancelin, Dany Severac, Gilles Clément, Pascal Tillard, Florian Frugier, Marc Lepetit

**Affiliations:** ^1^ Université Paris-Saclay, CNRS, INRAE, Univ d’Evry, Institute of Plant Sciences Paris-Saclay (IPS2), Gif sur Yvette, France; ^2^ Université Paris Cité, Institute of Plant Sciences Paris-Saclay (IPS2), Gif sur Yvette, France; ^3^ Université Paris-Saclay, AgroParisTech, INRAE, UMR MIA, Paris-Saclay, Palaiseau, France; ^4^ LSTM, Laboratoire des Symbioses Tropicales et Méditerranéennes, INRAE, IRD, CIRAD, Institut Agro Montpellier, Université de Montpellier, Montpellier, France; ^5^ PHIM Plant Health Institute, INRAE, Université de Montpellier, CIRAD, Institut Agro, IRD, Montpellier, France; ^6^ MGX, CNRS, INSERM, Université de Montpellier, Montpellier, France; ^7^ Institut Jean-Pierre Bourgin, INRAE, AgroParisTech, CNRS, Université Paris-Saclay, Versailles, France; ^8^ Biologie et Physiologie Moléculaire des Plantes, INRAE, CNRS, Institut Agro Montpellier, Université de Montpellier, Montpellier, France; ^9^ Institut Sophia Agrobiotech, INRAE, CNRS, Université Côte d'Azur, Sophia-Antipolis, France

**Keywords:** symbiosis, nitrogen, osmotic stress, systemic signaling, *Medicago truncatula*, *Rhizobium*

## Abstract

In mature symbiotic root nodules, differentiated rhizobia fix atmospheric dinitrogen and provide ammonium to fulfill the plant nitrogen (N) demand. The plant enables this process by providing photosynthates to the nodules. The symbiosis is adjusted to the whole plant N demand thanks to systemic N signaling controlling nodule development. Symbiotic plants under N deficit stimulate nodule expansion and activate nodule senescence under N satiety. Besides, nodules are highly sensitive to drought. Here, we used split-root systems to characterize the systemic responses of symbiotic plants to a localized osmotic stress. We showed that polyéthylène glycol (PEG) application rapidly inhibited the symbiotic dinitrogen fixation activity of nodules locally exposed to the treatment, resulting to the N limitation of the plant supplied exclusively by symbiotic dinitrogen fixation. The localized PEG treatment triggered systemic signaling stimulating nodule development in the distant untreated roots. This response was associated with an enhancement of the sucrose allocation. Our analyses showed that transcriptomic reprogramming associated with PEG and N deficit systemic signaling(s) shared many targets transcripts. Altogether, our study suggests that systemic N signaling is a component of the adaptation of the symbiotic plant to the local variations of its edaphic environment.

## Introduction

Legumes have the unique ability to associate with rhizobia in symbiotic root organs called nodules. Within nodules, differentiated bacteroids use the dinitrogen (N_2_) from the air as an unlimited nitrogen (N) source (Oldroyd et al., 2011; Roy et al., 2020). The bacterial nitrogenase converts atmospheric N_2_ into ammonium, which will be assimilated into amino acids by the plant cells. In return, the plant fuels the symbiotic N_2_ fixation (SNF) by providing products of shoot photosynthesis to the bacteria. Therefore, the carbon metabolite flux from the shoots to the bacteroids and the nitrogenase activity in the root nodules are necessarily tightly correlated (Udvardi and Poole, 2013).

Although symbiosis may allow the plant to circumvent the soil mineral N limitation, N fixation is highly dependent on soil conditions, particularly on abiotic stresses such as drought. In soybean nodules, the nitrogenase activity declines very sharply when the nodule water potential decreases (Huang et al., 1975; Durand et al., 1987). This decline in response to drought occurs before any effect on photosynthesis is detected (Durand et al., 1987). Local drought applied in *Medicago truncatula* plants grown in split-root systems showed that the inhibition of the nitrogenase activity by drought is local, i.e., only in nodules directly exposed to drought (Gil-Quintana et al., 2013a; Gil-Quintana et al., 2013b). This inhibition is associated with a decline of the nodule water potential but occurs before any decrease of leaf water potential and of plant evapotranspiration. Such local drought applied on a half root system has little effect on the whole plant water status because of the compensation by the distant watered roots, enabling the water supply of the whole plant. Earlier studies have proposed several hypotheses to explain the local inhibition of water limitation on SNF, but they remain to be validated (Huang et al., 1975; Durand et al., 1987; Marino et al., 2007; Gil-Quintana et al., 2013a; Gil-Quintana et al., 2013b).

Symbiotic nodule development and functioning are dependent on the whole plant N status and, therefore, highly dependent on its N acquisition capacities (Lepetit and Brouquisse, 2023). N-related systemic signaling regulations between shoots and roots were investigated using split-root systems in symbiotic *M. truncatula* plants (Ruffel et al., 2008; Jeudy et al., 2010; Laguerre et al., 2012; Lambert et al., 2020b; Pervent et al., 2021). These systemic regulations are instrumental for optimizing the root N “foraging” behavior, which orchestrates underground organ growth in soil conditions that are heterogeneous in time and space, including for the nutritional resources. Following the early interaction of *Rhizobium* with legume host roots, nodule formation requires plant N deficit and is inhibited by plant N satiety (Pervent et al., 2021). Whole plant N satiety systemic signaling rapidly represses the N_2_ fixation activity of mature nodules and initiates their senescence (Lambert et al., 2020b). In split-root systems, a whole plant N deficit under symbiotic conditions can be obtained on a half root system either by a local treatment substituting air by Ar/O_2_ (80/20 v/v) or by inoculating the root with non-N_2_-fixing (fix^-^) mutant bacteria (Laguerre et al., 2012). Both treatments provoke a whole plant N deficit and trigger a systemic response in the distant untreated half root system, stimulating the expansion of preexisting nodules and the formation of additional symbiotic organs (Jeudy et al., 2010; Laguerre et al., 2012). This N-deficit systemic signaling enables the plant to compensate for a lower N_2_ fixation in the inefficient part of its symbiotic root system by stimulating nodule development and, consequently, SNF in the compensatory roots. In symbiotic plants that are N supplied exclusively through SNF, rapid nodule sucrose content variations were associated with systemic N signaling, suggesting that a sugar allocation toward nodules may be involved in the signaling process (Jeudy et al., 2010; Lambert et al., 2020b). Transcriptome reprogramming in response to systemic N signaling has been characterized in roots of *M. truncatula* during the early stages of the interaction with *Sinorhizobium medicae* and in mature differentiated N_2_-fixing nodules of plants grown in split-root systems (Lambert et al., 2020b; Pervent et al., 2021). Many of the genes involved in nodule formation are strongly regulated by systemic N signaling, consistently with the plant N deficit being mandatory for symbiosis establishment (Pervent et al., 2021). Molecular mechanisms involved in the systemic control of nodule formation have been discovered, involving shoot receptors belonging to the CLAVATA1 family to perceive signaling peptides of the CLE and CEP families (Gautrat et al., 2021; Roy and Müller, 2022). How these components contribute to the regulation of nodulation by N signaling remains however a challenging question (Gautrat et al., 2021; Lepetit and Brouquisse, 2023). Less is known about mechanisms responsible for the regulation of mature nodules by systemic N signaling. The transcriptomic responses of mature nodules to N-satiety and N-deficit systemic signaling were recently characterized using *M. truncatula* plants grown in split-root systems (Lambert et al., 2020b). The N-satiety systemic signaling response includes the rapid activation of a nodule senescence program and the repression of many transcripts associated with nodule development and functioning. The N-deficit signaling response involves a large transcript reprogramming mainly related to cell division, bacteroid differentiation through the function of NCR and GRP peptides, as well as sugar transport, leghemoglobin accumulation, and specific hormonal responses (Lambert et al., 2020b). Although transcript reprogramming has been characterized, pathways responsible for systemic N signaling in mature nodules remains not yet unraveled.

In this study, we aimed to characterize the systemic response of symbiotic plants to a local osmotic constraint. *M. truncatula/S. medicae* symbiotic plants with mature nodules were grown hydroponically in split-root systems. We included Polyéthylène glycol (PEG) in the medium to apply the osmotic constraint (Zahran and Sprent, 1986; Couchoud et al., 2019). PEG was added to half of the roots, and the systemic effects of this treatment were investigated on the remaining distant untreated roots. The SNF, nodule sucrose content, plant growth, and transcriptome reprogramming of mature nodules in response to systemic PEG signaling were compared to the previously characterized response to systemic N signaling (Lambert et al., 2020b). These combined approaches allowed highlighting the implication of systemic N responses in the symbiotic plant adaptation to a localized environmental fluctuation.

## Materials and methods

### Split-root plant growth conditions

**Figure 1:**
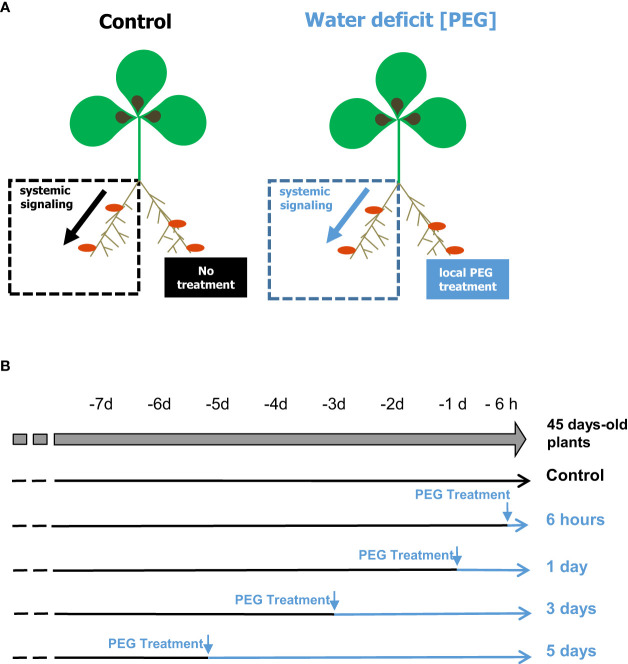
Split-root systems used to compare whole plant responses to localized PEG treatments. Nodulated plants were cultivated hydroponically on a nutrient solution containing no mineral N. PEG treatments were applied on a half root system; the other part of the roots being maintained in initial conditions. Local responses to the treatments were investigated by comparing the treated and the distant nontreated roots of the same plant. **(A)** Systemic responses to the treatments were investigated by comparing the nontreated roots maintained in the same local environment (dash square) of plants cultivated under different regimes. **(B)** Shoot, nodules, or denodulated roots were collected simultaneously on plants of the same age that have been treated for various durations by PEG or maintained without treatment.

The *M. truncatula* genotype A17 was grown hydroponically in split-root systems as described by Lambert et al. (2020b). Three-week-old plants were inoculated with *S. medicae* md4 bacteria (10^7^ CFU/mL). An HY nutrient solution (Lambert et al., 2020b), not supplemented with mineral N and adjusted to pH7, was used and renewed every week. The root systems of 5-week-old plants were separated into two parts, each side being installed in a compartment of the split-root experimental system (**Figure 1A**). PEG treatment conditions (150 g/L PEG8000) were deduced from previous *M. truncatula* A17 studies (Couchoud et al., 2019) and locally applied on one side of the root system, while initial growth medium conditions were maintained on the other half root system. Nutrient solutions of each compartment were renewed daily. Roots were harvested from 45-day-old plants (**Figure 1B**). ^15^N_2_ fixation and N intake measurements were done on excised roots according to Ruffel et al. (2008). The method uses short-term labeling to circumvent the potential bias associated with carbon metabolite starvation of excised roots. Freshly excised nodulated roots were placed in airtight 10-mL tubes containing 2 mL of basal nutrient solution. Ten minutes of labeling was achieved by replacing in each tube 5 mL of air with 5 mL of 80% ^15^N_2_/20% O_2_ mix (99 atom% ^15^N). Samples (100 µL) of ^15^N_2_-enriched air were harvested at the beginning and end of the labeling for precise analysis of the atom% ^15^N of the ^15^N_2_ source and leak check. After labeling, nodules were separated from roots, and both organs were collected, dried at 70°C for 48 h, weighed, and analyzed for total ^15^N content using a continuous-flow isotope ratio mass spectrometer (Isoprime mass spectrometer; GV Instruments) coupled to a nitrogen elemental analyzer (EuroVector S.p.A.). Ruffel et al. (2008) reported that measurements of ^15^N_2_ fixation on excised nodulated roots in this condition were equivalent to those obtained on intact plant roots. Analyses of nodule metabolite contents by GC/MS were achieved, as described in Lambert et al. (2020b).

### RNA sequencing sample collection and preparation

Each sample was a pool of nodules collected from five plants. Three replicate samples were generated for each biological condition. After nodule grinding in liquid nitrogen, RNAs were extracted using the miRNeasy^®^ Mini Kit (Qiagen, Hilden, Germany) according to the supplier’s recommendations. An RNA quality control was performed on a Bioanalyzer (BioRad Laboratories Inc., Hercules, CA, USA). Polyadenylated plant mRNA libraries were prepared using the TruSeq Stranded mRNA Sample Preparation Kit (Illumina, San Diego, CA, USA). Library quality was checked using the Standard Sensitivity NGS Kit on an Advanced Analytical Fragment Analyzer (Agilent Technologies, Inc., Santa Clara, CA, USA). Clustering and primer hybridizations were performed both on the Illumina HiSeq 2500, using the TruSeq Rapid Cluster Kit, or on the Illumina cBot (Illumina, San Diego, CA, USA). Sample sequencing was achieved in the single-read 50-nucleotide mode using the Sequence By Sequencing (SBS) technology on the Illumina HiSeq 2500 with the TruSeq Rapid SBS Kit according to supplier instructions, consumables, and software. Sequencing quality controls were performed using the FastQC and FastQ Screen open-access software (Babraham Institute, Cambridge, UK) to test for contaminations by unexpected organisms. RNA sequencing (RNAseq) data are available on ArrayExpress under the ID number E-MTAB-10497 (https://www.ebi.ac.uk/arrayexpress/experiments/E-MTAB-10497/).

### RNAseq data analysis

Sequencing reads were mapped to the MtruncA17r5.0 latest version of the *M. truncatula* genome (Pecrix et al., 2018) using the glint software (http://lipm-bioinfo.toulouse.inra.fr/download/glint/). An alignment of a minimum length of 40 nucleotides with less than three mismatches and three gaps was allowed. Reads mapping to different genomic positions were omitted. The statistical analysis was performed in the R version 3.5.1 using the DiCoExpress tool (Lambert et al., 2020a; Baudry et al., 2022) that uses EdgeR (v.3.22.3) and tCoseq (v.1.4.0) Bioconductor packages. The counts per million method was used with a threshold of one read per million in half of the samples. Libraries were normalized using the Trimmed Mean of M-values method (Robinson et al., 2010). Differential analysis was performed using a negative binomial generalized linear model where the log of the average gene expression was considered as a replicate effect plus a treatment effect (Control and 6 h, 1 day, 3 days, and 5 days of PEG treatment). A likelihood ratio test was performed to evaluate the expression changes between two consecutive durations of the PEG treatment (no treatment being the zero duration), and P-values were adjusted with the Benjamini–Hochberg procedure to control the false discovery rate (FDR). An adjusted P-value lower than 0.05 was considered to define differentially accumulated transcripts (DATs). For the co-expression analysis (Coseq package), the component number in the mixture was chosen according to the integrated completed likelihood (ICL) criterion. We implemented the MtruncA17V4.2 annotations, the manual annotations generated in Lambert et al. (2020b), the plant metabolic network (PMN)-MedicCyc annotations (Urbanczyk-Wochniak and Sumner, 2007), the symbiotic island annotations (Pecrix et al., 2018), and the Mapman MtruncA17V4.0 annotations (Tellström et al., 2007) into the MtA17 r5.0 gene annotation, but only when an unequivocal correspondence confirmed by blast was evidenced. The enrichment analysis for a given annotation term was performed using a hypergeometric test. To control the FDR among all tested terms, raw P-values were adjusted by the Benjamini–Hochberg procedure, and enriched terms were defined as those having an adjusted P-value lower than 0.05. Using this procedure, we tested if specific biological functions were enriched in annotations of DATs compared to their abundance in the nodule transcriptome. The global Gene Ontology (GO) analysis of DATs, using the whole *M. truncatula* genome as the reference, was performed with the BiNGO-Cytoscape plugin (Maere et al., 2005), which also uses a hypergeometric test and the Benjamini–Hochberg procedure to adjust P-values.

## Results

### PEG suppresses N fixation locally and is associated with systemic signaling at the whole plant level

**Figure 2:**
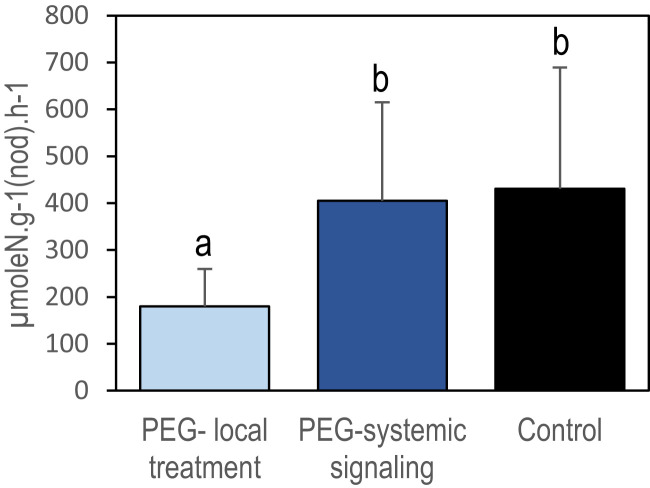
Local repression of specific nitrogen fixation activity by local PEG treatment in split-root systems. Nitrogen fixation activity was measured after 48 h of PEG treatment using ^15^N_2_ labeling on excised nodulated root systems. Blue and black bars referred respectively to the PEG-treated and control plants (**Figure 1**). The light blue bar represents the nodules of the half root system directly exposed to PEG and the dark blue bar represents the nodules of the other untreated half root system (i.e., not directly exposed to the treatment). Values are mean ± SD, n = 8. Letters indicate distinct groups of values deduced from Kruskal–Wallis and pairwise Wilcoxon tests using a P-value threshold of 0.05 and Benjamini–Hochberg correction for multiple testing.

**Figure 3:**
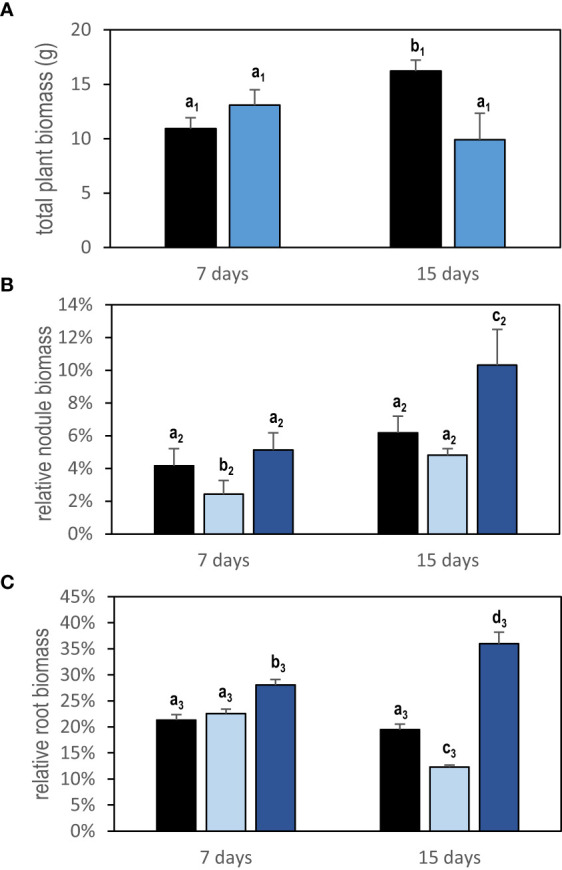
Growth response to local PEG treatment of plant cultivated in split-root systems. Plants were grown in a split-root system equivalent to that in **Figure 1** except that the PEG treatment was applied on 40-day-old plants for 7 days (47-day-old plants) or 15 days (55-day-old plants). Control plants were cultivated in parallel in the same conditions without treatment. **(A)** Total plant biomass of PEG-treated (blue bars) and control plant (black bars). **(B)** Relative nodule biomass of half root systems (half root system nodule biomass/total plant biomass). **(C)** Relative root biomass of half root systems (half root system root biomass/total plant biomass). The light blue bars represent the half root systems directly exposed to PEG, and the dark blue bars represent the distant half root systems not directly exposed to the treatment but under the control of systemic signaling. Black bars represent the control roots. Values are mean ± SD, n = 5. Letters indicate distinct groups of values deduced from Kruskal–Wallis and pairwise Wilcoxon tests using a P-value threshold of 0.05 and Benjamini–Hochberg correction for multiple testing.

Localized osmotic stress (PEG treatment) was applied to *M. truncatula* plants with N_2_-fixing nodules formed upon inoculation with the *S. medicae* md4 strain. Symbiotic plants were grown hydroponically in an aerated nutrient solution without mineral N and thus N supplied exclusively through SNF. Symbiotic root systems were separated into two compartments containing half root systems, only one of them being treated with PEG (**Figure 1A**). The local and systemic effects of the localized PEG treatment on N intake were analyzed in both sides of the root system after a 48-h treatment. A drastic reduction of the specific SNF activity (N_2_ fixation per nodule biomass) was measured in the half root system directly exposed to the treatment, whereas the SNF activity was not affected in the distant half root system not directly exposed to PEG (**Figure 2**). The 48-h local PEG treatment did not result in a significant change in plant organ fresh biomasses. The biomass of shoots, denodulated half root systems, and their corresponding nodules was respectively shoot_FW_ = 5.45 ± 0.92 g, roots_FW_ = 2.35 ± 0.65 g, and nodules_FW_ = 0.42 ± 0.10 g. Based on the N fixation measurements, we calculated the N intake driven by each half root system and by whole plants (treated and control). In control plants, the calculated whole plant N intake was 360.92 µmoleN.h^-1^, whereas in treated plants, it was 245 µmoleN.h^-1^, displaying an almost 50% reduction. Because in symbiotic *M. truncatula* plants exclusively relying on N_2_ fixation, the N acquisition was generally limiting plant growth (Moreau et al., 2008; Ruffel et al., 2008; Laguerre et al., 2012), we concluded that PEG-treated plants experienced an N deficit as compared to control plants. Plant growth was monitored for 7 and 15 days (**Figure 3**). The PEG treatment resulted in a growth reduction of the whole plant after 15 days likely related to the plant N deficit associated with the treatment (**Figure 3A**). We analyzed the relative growth of the plant organs by normalizing their biomass relative to the whole plant biomass. In the half root system locally exposed to PEG, the nodule and root relative growth was reduced as compared to the untreated side (**Figures 3B, C**). Conversely, in the distal half root systems of the same plants that were not directly exposed to the PEG treatment, a stimulation of nodule and root relative growth was observed (**Figures 3B, C**). After 15 days of treatment, the root and nodule relative biomasses of the untreated distant half roots of PEG-treated plants were significantly higher than those in the half root systems of control plants. These differential growth stimulations resulted in the ability of the whole plant to compensate for the negative impact of local stress. Accordingly, the PEG-treated plant allocated less biomass to the PEG-treated roots to the benefit of the untreated roots. Because the untreated roots of the PEG-treated and of the control plants shared the same local environment, their compensatory responses were unambiguously explained by the occurrence at the whole plant level of a systemic signaling. Interestingly, similar conclusions could be reached with split-root plants grown on sand and exposed to local drought (**Supplementary Figure S1**) or with split-root plants grown hydroponically and exposed locally to 75-mM NaCl stress (**Supplementary Figure S2**). In both systems, systemic signaling compensatory responses to the local constraints were observed, leading to the stimulation of both nodule and root growth in systemic roots not directly exposed to the local treatments. We thus concluded that the observed root and nodule growth compensatory systemic responses were not specific to the PEG treatment and could be generalized to other local water constraints resulting from drought, PEG, or NaCl treatments.

**Figure 4:**
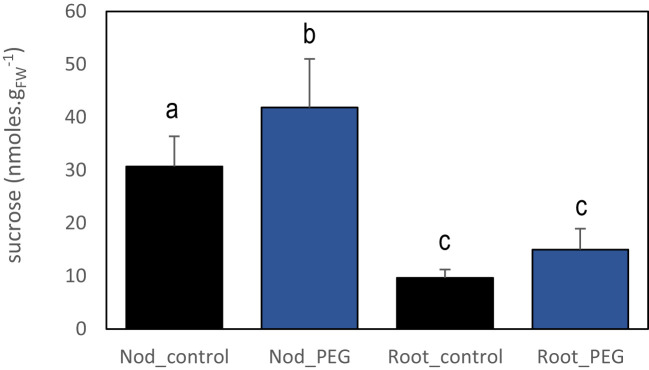
Effect of systemic PEG signaling on nodule and root sucrose contents. PEG-treated plants for 3 days were cultivated in split-root systems described in **Figure 1**. The sucrose content was quantified in roots and nodules not directly exposed to PEG of PEG-treated plants and control plants. Values are mean ± SD, n = 4. Letters indicate distinct groups of values deduced from Kruskal–Wallis and pairwise Wilcoxon tests using a P-value threshold of 0.05 and Benjamini–Hochberg correction for multiple testing.

The reduced SNF activity provoked by the local PEG treatment resulted in a transient reduction of whole plant N acquisition and associated with a systemic response stimulating nodule development. We thus hypothesized that the systemic compensatory response to a localized PEG osmotic stress treatment was, at least in part, the result of the activation of a plant N-deficit systemic signaling. In previous studies, rapid changes in carbon metabolite allocation from the shoots to the nodules were associated with systemic N signaling (Jeudy et al., 2010; Lambert et al., 2020b). We then tested sucrose allocation in response to local PEG treatment, revealing that the associated systemic signaling also increased sucrose content in nodules after 3 days of local PEG treatment (**Figure 4**).

### Systemic PEG and N signaling share numerous nodule transcriptomic targets

The plant transcriptomic responses of mature N_2_-fixing nodules of *M. truncatula* plants, inoculated with *S. medicae md4*, to systemic PEG signaling were investigated using the split-root systems described in **Figure 1A**. Nodules of the distant untreated roots of the PEG-treated plants were collected after 6 h, 1 day, 3 days, or 5 days of treatment (**Figure 1B**). Nodules of untreated plants grown in parallel split roots were used as controls. RNAseq analysis was performed on total RNAs, and quality control analyses are provided in **Supplementary Figure S3**. Differential analyses allowed identifying a large transcriptome reprogramming occurring in nodules in response to PEG systemic signaling (**Supplementary Figure S4**). A total of 10,904 DATs were identified in at least one of the pairwise comparisons between control, 6 h, 1 day, 3 days, and 5 days (PEG_DATs; **Supplementary Table S1**). Analysis of GO terms corresponding to all of these transcripts using the BiNGO package revealed that GO terms related to “response to stress” and to “nodule morphogenesis,” “nodulation,” and “symbiosis” were overrepresented in systemic PEG_DATs as compared to the whole genome (**Supplementary Figure S5**), in agreement with the physiological impact of systemic PEG signaling on nodule development.

**Figure 5:**
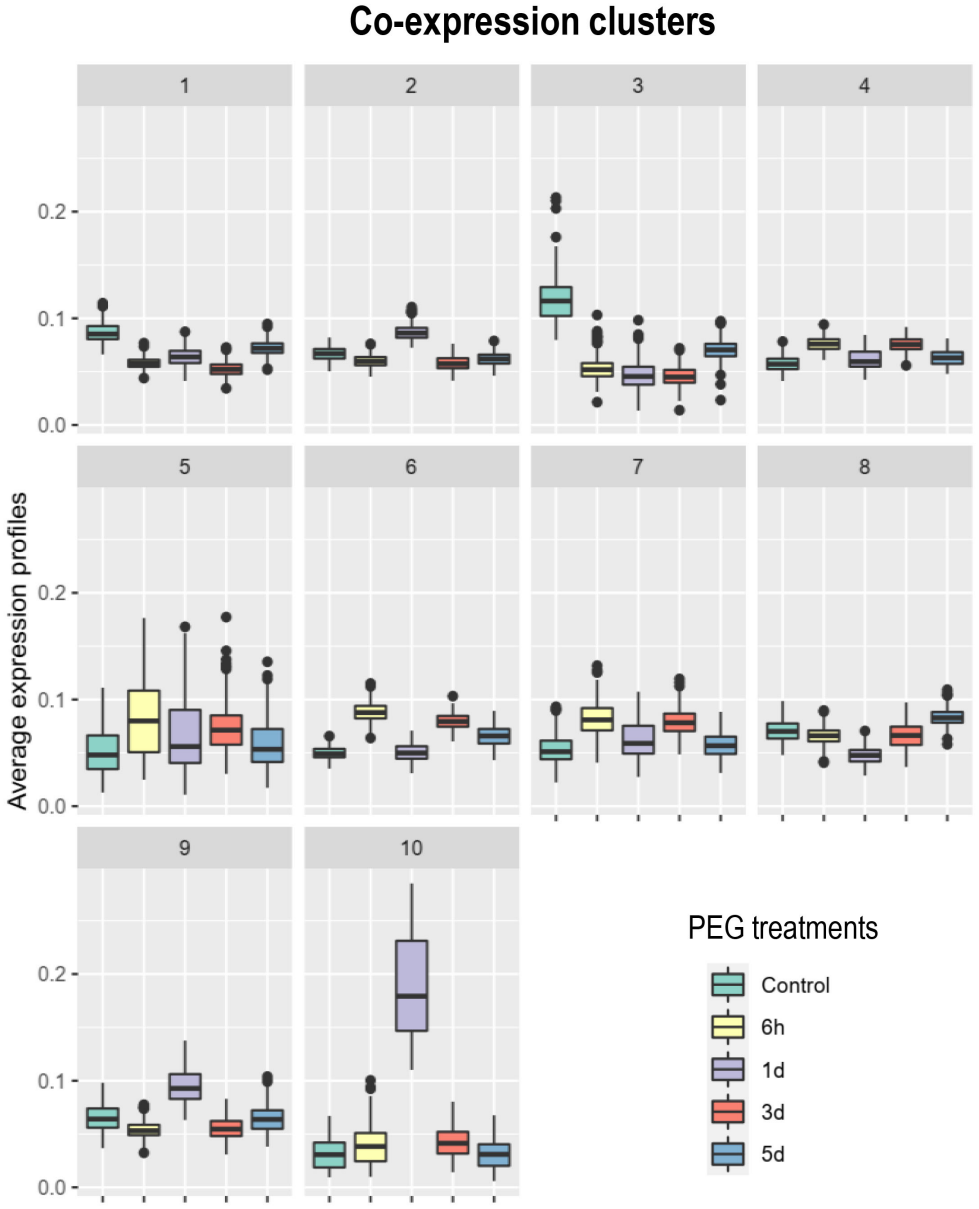
Co-expression analysis of the transcripts differentially accumulated in nodules in response to systemic N and PEG signaling (N&PEG DATs). Plants were cultivated in split-root systems described in **Figure 1**. Root transcripts were analyzed by RNAseq at 6 h, 1 day, 3 days, and 5 days after the initiation of the PEG treatment. Co-expression clusters and their normalized accumulation kinetics were identified using the Coseq package of the DiCoExpress platform. N&PEG DATs, specified co-expression clusters, gene names, and annotations are listed in **Supplementary Table S2**.

To test the hypothesis of recruitment of systemic N signaling during the PEG-induced systemic transcriptomic reprogramming, we investigated if the transcripts responsive to systemic N signaling in mature nodules that were identified in a previous study using the same biological system (Lambert et al., 2020b) were particularly abundant in PEG_DATs. Most (4,277 DATs; 57%) of the transcripts responsive to systemic N signaling were retrieved in the PEG_DATs and, therefore, called N&PEG DATs (**Supplementary Table S2**). Hypergeometric tests, using the whole nodule transcriptome as a reference, confirmed this strong relative enrichment of N&PEG_DATs within the PEG_DATs (**Table 1**). Having identified this striking overlap, further analyses were then focused specifically on N&PEG_DATs. A co-expression analysis based on mixture models (Coseq package) organized these N&PEG_DATs according to their accumulation kinetics in 10 co-expression clusters (**Figure 5**, **Supplementary Table S2**). The model fitted the data well, as only less than 10% of the transcripts were not classified (cluster 0; **Supplementary Table S2**). We compared these systemic PEG signaling nodule co-expression clusters to the systemic N signaling nodule response clusters identified by Lambert et al. (2020b). The relative distribution of transcripts in the two cluster datasets was not random, arguing that both responses to systemic PEG signaling and to systemic N signaling were not independent (**Supplementary Table S3**). The metacluster A, defined by Lambert et al. (2020b), contained transcripts mainly retrieved in N&PEG_DAT clusters 2, 5, 9, and 10, whereas the metacluster B contained transcripts mainly retrieved in N&PEG_DAT clusters 1, 3, 4, 6, and 8.

**Table 1:** Overlaps between nodule transcriptome responses to systemic N signaling and PEG signaling.

Term	Transcript numbers	Proportion in PEG_DATs %	Enrichment FDR
SN_2_ in PEG	3,838	56	7.63.10^-90^
DN_2_ in PEG	1,465	66	8.22.10^-86^
(DN_2_ or/and SN_2_) in PEG	4,277	57	1.04.10^-110^

The total number of significantly expressed nodule transcript detected in this study was 23,610. Among them, differentially accumulated transcripts in response to PEG were 10,904 (PEG_DATs). The DN_2_ and SN_2_ mature nodule transcripts, responding respectively to systemic signaling of N Deficit and N Satiety, identified by Lambert et al. (2020b) were assigned to the MtruncA17r5.0 annotation by blast (1,465 and 3,838 transcripts, respectively). Specific enrichments of these DN_2_ and/or SN_2_ transcripts in the PEG_DATs list were evaluated using hypergeometric tests relative to all accumulated transcripts of the organ as a reference. Enrichment was declared when the false discovery rate (FDR) associated with the hypergeometric test was lower than 0.05.

**Figure 6:**
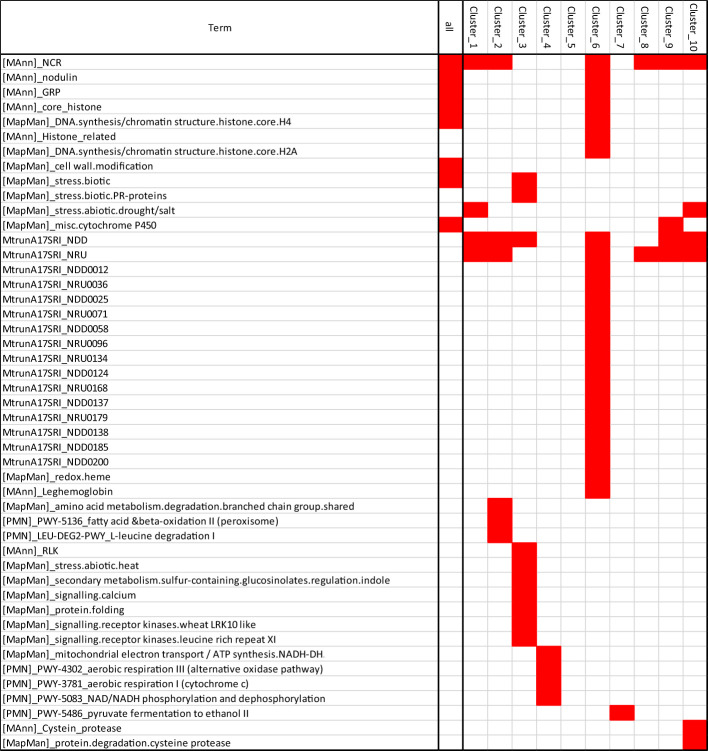
Enrichment in specific biological function annotations of N&PEG DATs co-expression clusters. Red blocks indicate specific annotation enrichment of the cluster as compared to the nodule transcriptome (estimated by hypergeometric test with a 0.05 adjusted P-value threshold). Annotation terms are those of the MtruncA17V4.2 (gene annotation and associated manual class annotation; Lambert et al., 2020b), the MtruncA17r5 with symbiotic island Symbiosis Related Islands (SRIs) annotation of Pecrix et al. (2018), the Plant Metabolic Network MedicCyc annotation (PMN Pathway name), and the Mapmann/MtruncA17V4.0 annotation (Tellström et al., 2007).

The analysis of N&PEG_DAT functional annotations (**Figure 6**) revealed in several co-expression clusters specific enrichments for transcripts belonging to the *M. truncatula* “symbiotic islands” identified by Pecrix et al. (2018), namely, NRUs (Nodule vs. Root Upregulated) and NDDs (Nodule Development and Differentiation), confirming the particular impact of both PEG and N systemic signalings on the symbiosis. More specifically, transcripts encoding NCR peptides (**Supplementary Figure S6**), GRP peptides (**Supplementary Figure S7**), core histones (**Supplementary Figure S8**), leghemoglobins (**Supplementary Figure S9**), SWEET sucrose transporters (**Supplementary Figure S10**), and nodule-associated transcripts (annotated as “nodulins”) were overrepresented in N&PEG_DATs. This further documented at the transcriptome level the common features of systemic PEG and N signaling responses previously highlighted at the physiological level. Among N&PEG_DATs were notably retrieved transcripts related to the symbiotic recognition of rhizobia, to the Nod factor signaling pathway, and to symbiotic infection (MtNPL, MtRPG, MtLYK10, MtDMI3, MtNSP2, MtEFD, and MtEFD2; **Supplementary Table S2**), indicating that bacterial infections are likely affected by both systemic PEG and N signaling.

## Discussion

This work illustrated the tight integration of the *Rhizobium*–legume symbiotic interaction with the whole plant nutritional regime and gave new insight into its biological significance. Integration involves systemic signaling to adjust the root symbiotic capacity to the nutritional demand of the plant as a function of the whole plant growth (Lepetit and Brouquisse, 2023). These split-root studies revealed compensatory responses to the local PEG treatment tending to maintain the whole symbiotic activity through inter-organ signaling. Distant responses resulting from the local PEG treatment applied on a half root system were observed in the untreated half root system of the plant. Indeed, both local treatments resulted in the distant stimulation of nodule development, allowing compensation for the plant N deficit provoked by the local constraints. Therefore, systemic signaling was necessarily involved. The systemic PEG signaling response shared many features with a previously documented systemic response provoked by plant N status variation (Lambert et al., 2020b), suggesting that an N signaling component was shared between both systemic responses.

In this study, local inhibition of symbiosis by PEG, NaCl, or drought resulted in an inhibition of SNF and in plant N limitation. Using ^15^N_2_ labeling on intact split-root plant as described by Jeudy et al. (2010) might rule out any possible bias due to root excision, since the latter may affect the oxygen diffusion barrier as described by Minchin et al. (1986). Nevertheless, our results are consistent with previous reports. Local inhibition of SNF by drought was previously demonstrated in *M. truncatula* and soybean plants grown in split-root systems and exposed to localized drought (Gil-Quintana et al., 2013a; Gil-Quintana et al., 2013b). We confirmed this local inhibitory effect in *M. truncatula* using PEG or NaCl treatments affecting the osmotic potential of the medium. Although these local treatments locally impair water acquisition, the systemic signaling associated with these treatments is unlikely related to whole plant water limitation. In split-root systems, local water limitation does not necessarily impact the whole plant’s water status because of the simultaneous maintenance of water acquisition in non-stressed roots that thus sustain the shoot water potential and plant evapotranspiration (Gil-Quintana et al., 2013a). Both in soybean and in *M. truncatula*, the decline of SNF in response to local water limitation occurs before significant effects on nodule water potential or on plant evapotranspiration (Gil-Quintana et al., 2013a; Gil-Quintana et al., 2013b). However, local inhibition of SNF by local water limitation results in a drastic reduction of the whole plant N acquisition due to the absence of an immediate compensation of the local reduction of SNF by untreated roots (**Figure 2**; Gil-Quintana et al., 2013a; Gil-Quintana et al., 2013b). Therefore, stimulation of nodule and root development in the compartment not directly exposed to the PEG, NaCl, or drought stress is probably more a response to the whole plant N limitation rather than to a hypothetical whole plant water limitation. As a matter of fact, the increase of nodule biomass observed in response to this stress systemic signaling tended to compensate, at the whole plant level, the local inhibition of SNF in nodules directly exposed to the stress. The question of the origin of the systemic signaling remains open, as our experiments did not identify whether the systemic signaling response to PEG was activated in the treated roots and/or in the shoots.

As SNF results in N acquisition by the plant, the N demand has a central role in the systemic control of symbiotic plants relying exclusively upon SNF. The whole plant N demand tightly controls symbiotic organ formation and development (reviewed in Lepetit and Brouquisse, 2023). Local environmental factors inhibiting SNF are expected to activate this control, even when the N resource (atmospheric N_2_) remains unlimited. According to this model, we showed that systemic PEG signaling mimicked N limitation systemic signaling. In symbiotic *M. truncatula* plants grown in split-root systems, local suppression of SNF by artificial Ar/O_2_ treatments or partial inoculation with non-fixing symbiont was shown to activate systemic N limitation signaling (Jeudy et al., 2010; Laguerre et al., 2012; Lambert et al., 2020b). The mature nodule response to plant N deficit was associated with an increased nodule sugar allocation correlated with the stimulation of nodule development (Lambert et al., 2020b). Similarly, as for systemic symbiotic N signaling, PEG systemic signaling also 1) favors sucrose allocation toward the nodule and 2) is associated with a transcriptome reprogramming of several thousands of nodule transcripts already identified as responsive to systemic N signaling (Lambert et al., 2020b). Therefore, these molecular data support that N limitation systemic signaling is a component of the plant systemic response to local PEG exposure, activating the nodule development compensatory response observed in roots not directly exposed to stress. However, although PEG systemic signaling and Ar/O_2_ systemic signaling share similar features and numerous target transcripts, a significant proportion of the systemic PEG signaling responsive transcripts were not identified as N responsive in Lambert et al. (2020b). Systemic PEG signaling cannot be thus reduced to an N limitation response but involves additional components likely more specifically related to the PEG treatment.

In the context of climate change, water limitation periods will be more frequent and soil moisture will be more heterogeneous and fluctuating than ever (Dusenge et al., 2019). This is a threat to agriculture and a challenge for plant sciences to select genotypes better adapted to these new constraints. Simultaneously, there is an urgent need to increase plant protein production, namely, from grain legumes, for human food because of its lower carbon footprint as compared to animal proteins (Goldstein and Reifen, 2022). In addition, atmospheric ambient CO_2_ increases, potentially modifying the conditions of plant C acquisition (Thompson et al., 2017). The potential of legumes in this context has been highlighted because, unlike cereals acquiring mainly nitrate as an N source, symbiotic N_2_-fixing legumes may benefit from elevated CO_2_ (Rogers et al., 2009). Changes in the equilibrium determining the symbiotic C/N trade-offs in legumes may indeed be an opportunity for the selection of new genotypes investing more resources in root development and able to adapt to a water deficit under these new environmental conditions (Palit et al., 2020) or having a better recovery of transient drought periods (Couchoud et al., 2020). Symbiotic plant breeding must therefore now consider that local and transient drought stresses may result not only in water stress but also in plant N deficit due to its dramatic impact on SNF and, consequently, may inhibit plant growth before any significant effect on the whole plant water status occurs. This study highlights the capacity of the whole plant to rapidly allocate more photosynthates to roots able to maintain N_2_ fixation and plant N nutrition, as compared to inefficient symbiotic roots, as a key factor in plant adaptation to local water stresses. This complex trait may be a promising target for agronomic selection in the future.

## Data availability statement

The datasets presented in this study can be found in online repositories. The names of the repository/repositories and accession number(s) can be found in the article/**Supplementary Material**.

## Author contributions

M-LM: Data curation, Funding acquisition, Investigation, Methodology, Software, Supervision, Validation, Writing – review & editing, Writing – original draft. MP: Data curation, Investigation, Methodology, Validation, Writing – review & editing. IL: Data curation, Investigation, Methodology, Software, Writing – review & editing. SC: Investigation, Methodology, Software, Writing – review & editing. MT: Investigation. DS: Investigation, Methodology, Writing – review & editing. GC: Investigation, Methodology. PT: Investigation, Methodology. FF: Funding acquisition, Project administration, Supervision, Writing – review & editing. ML: Conceptualization, Data curation, Formal Analysis, Funding acquisition, Investigation, Methodology, Project administration, Resources, Supervision, Validation, Writing – original draft, Writing – review & editing.
