# Supplementary material for: Localized osmotic stress activates systemic responses to N limitation in Medicago truncatula–Sinorhizobium symbiotic plants
Source: Front Plant Sci. 2023 Nov 20;14:1288070. doi: 10.3389/fpls.2023.1288070 (PMC10694431; doi:10.3389/fpls.2023.1288070)
Supplement: Supplementary file 1 [file DataSheet_1.zip › Supplementary Figures S1-10.PDF]

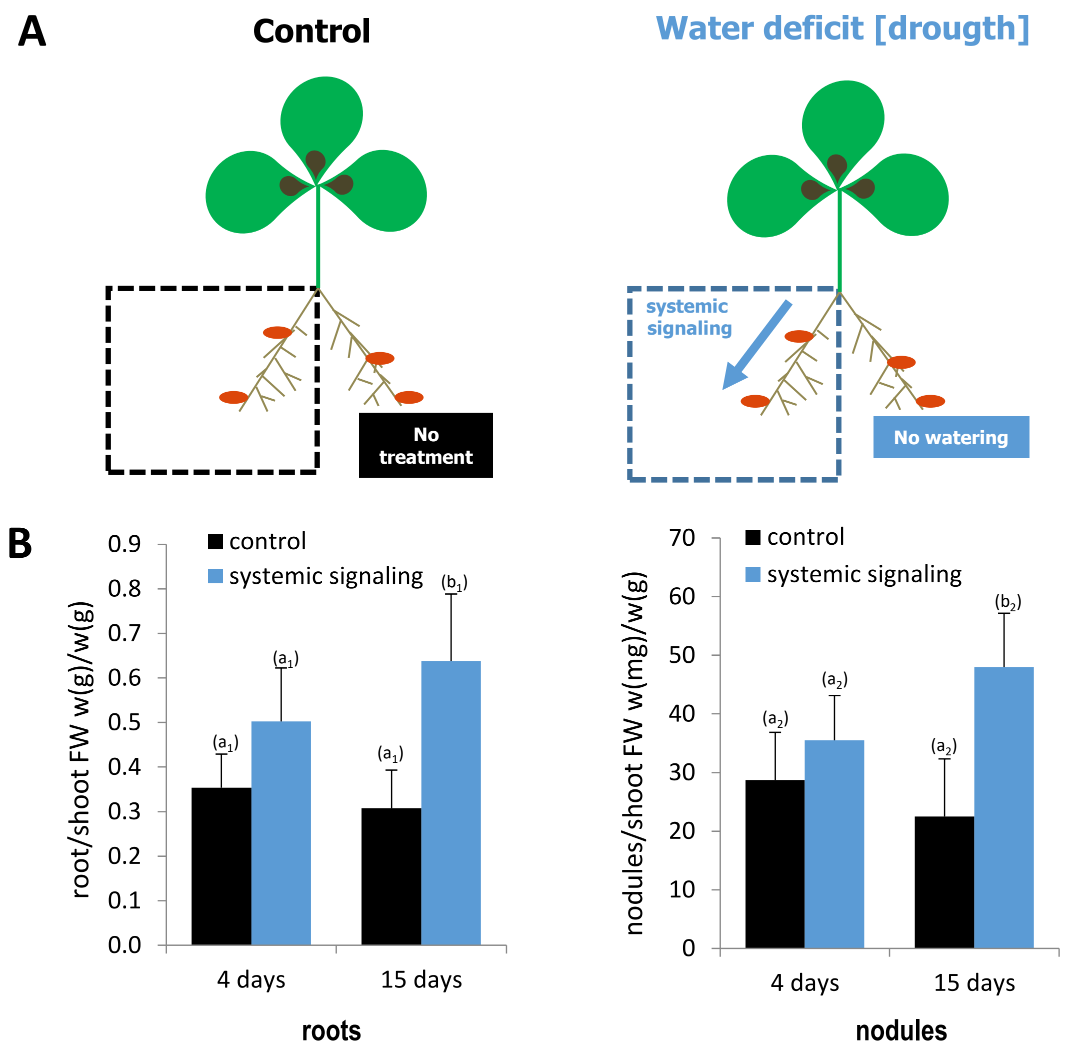

Supplementary Fig.S1. Systemic signaling associated to a local drought stress in *Medicago truncatula/Sinorhizobium medicae* symbiotic plants. A. Nodulated plants (21 days after inoculation) were grown in sand in split-root systems as described by Gil-Quintana et al (2012). Localized stress treatment consisted in stopping watering half of the root system (HY nutrient solution without mineral N). Soil water content was monitored during the following days using a radar TDR probe as humidimeter (IMKO HD2/SONO-M1, SDEC, France). Water limitation was effective when sand water content was below 3% w/w. Drought inhibited rapidly symbiotic N fixation in roots directly exposed to drought but not in distant roots (Gil-Quintana et al., 2012). B. The effect of systemic signaling on root and nodule growth was investigated by comparing distant watered half roots systems of plants submitted to the localized drought (blue bars), to half root systems of control plants (black bars) after 4 and 15 days of stress. Values are mean ± SD, n=5. Letters indicate distinct groups of values deduced from Kruskall-Wallis and pairwise Wilcoxon tests using a P-value threshold of 0.05, and Benjamini-Hochberg correction for multiple testing. FW, Fresh Weight.

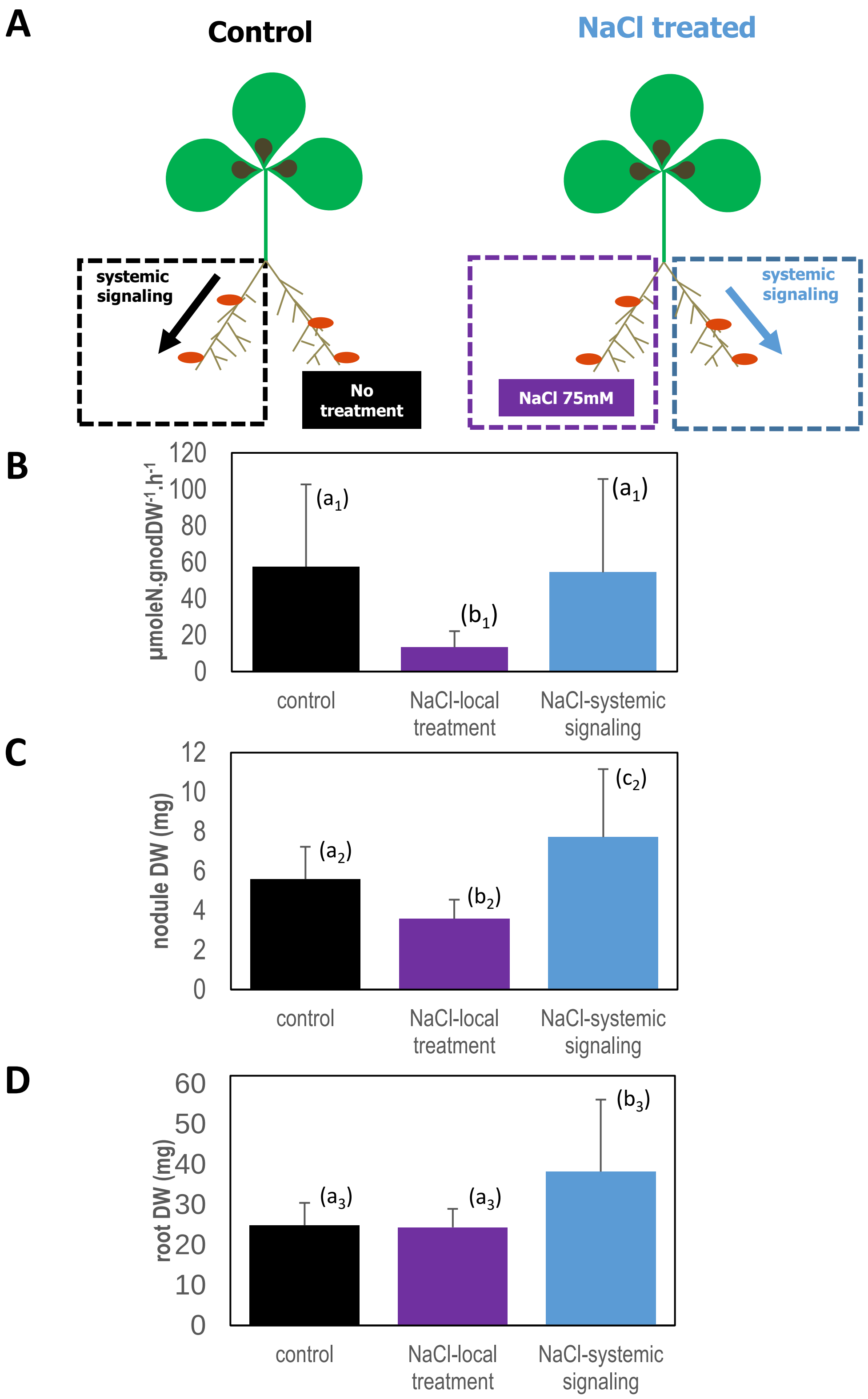

Supplementary Fig.S2. Systemic signaling associated to a local NaCl stress in *Medicago truncatula/Sinorhizobium medicae* symbiotic plants. A. Nodulated plants (21 days after inoculation) were grown hydroponically in split-root systems equivalent to those described in Fig1. The localized stress treatment consisted in adding Nacl 75mM to half of the root system (HY nutrient solution without mineral N) for 14 days. Local effect of NaCl addition was observed in the compartment directly exposed to the treatment (pink bars). The effect of systemic signaling associated to the treatment was investigated by comparing roots of control plants to the distant half root systems not directly exposed to the treatment but belonging to stressed plants (blue bars). Control plants were grown in split root systems without any NaCl treatment. B. $^{15}N_2$ fixation activity in roots of split-root systems compartments of plants after 5 days treatment. C. Biomass of nodules and roots of split-root systems compartments after 14 days of treatment. Values are mean ± SD, n=5. Letters indicate distinct groups of values deduced from Kruskall-Wallis and pairwise Wilcoxon tests using a P-value threshold of 0.05, and Benjamini-Hochberg correction for multiple testing. DW, Dry Weight.

A

B

C

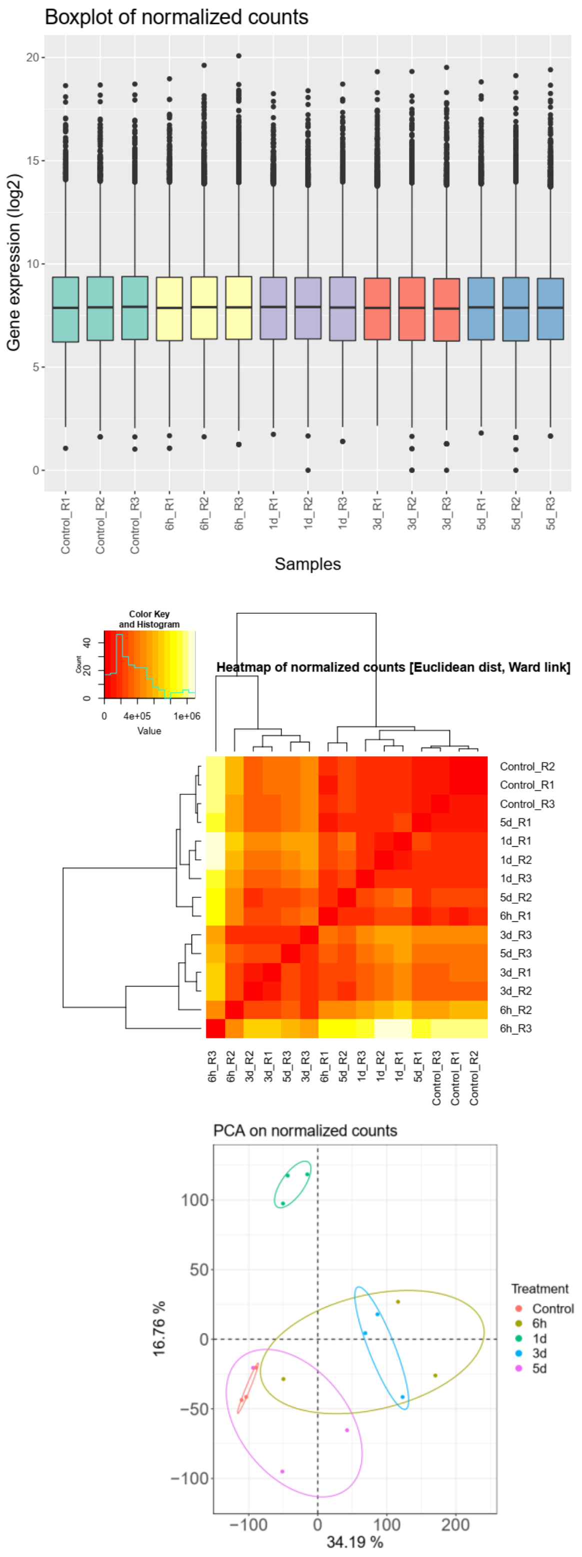

Supplementary Fig.S3. RNAseq data quality control. A Boxplots of normalized counts generated from all samples after different PEG-treatment durations: 6 hours (h), 1 day (d), 3d, 5d).. B Heatmap and hierarchical clustering of normalized counts generated from samples. C Principal component Analysis of normalized counts generated from samples. R1 to R3 are biological replicates.

A

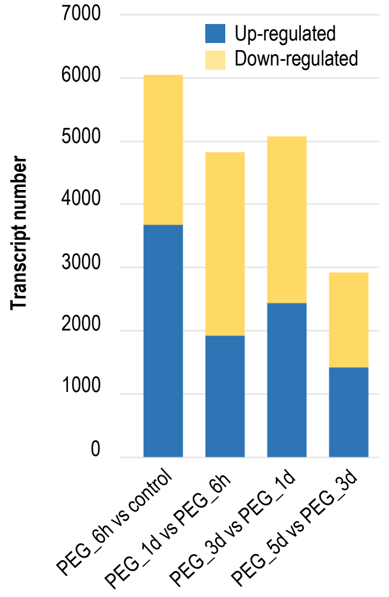

B

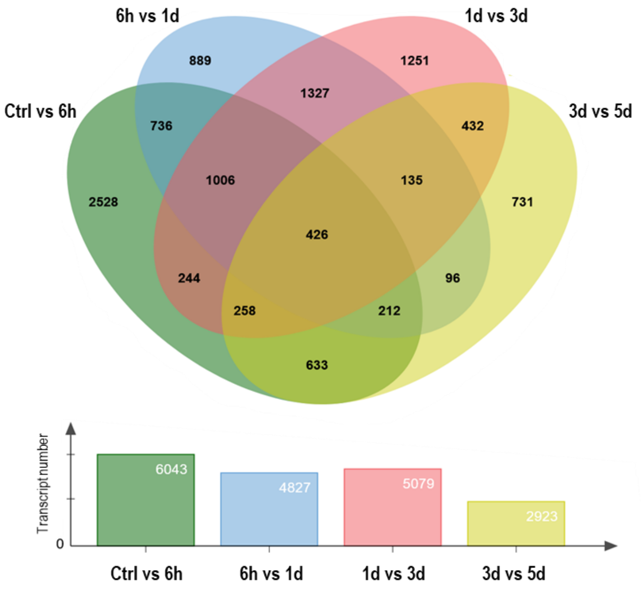

Supplementary Fig.S4. Differentially Accumulated Transcripts in response to systemic PEG signaling (PEG_DATs). A. Histogram of upregulated and down regulated PEG_DATs after different PEG-treatment durations: 6 hours (h), 1 day (d), 3d, 5d). B. Venn diagram of transcripts identified for different contrasts among the PEG-treatments.

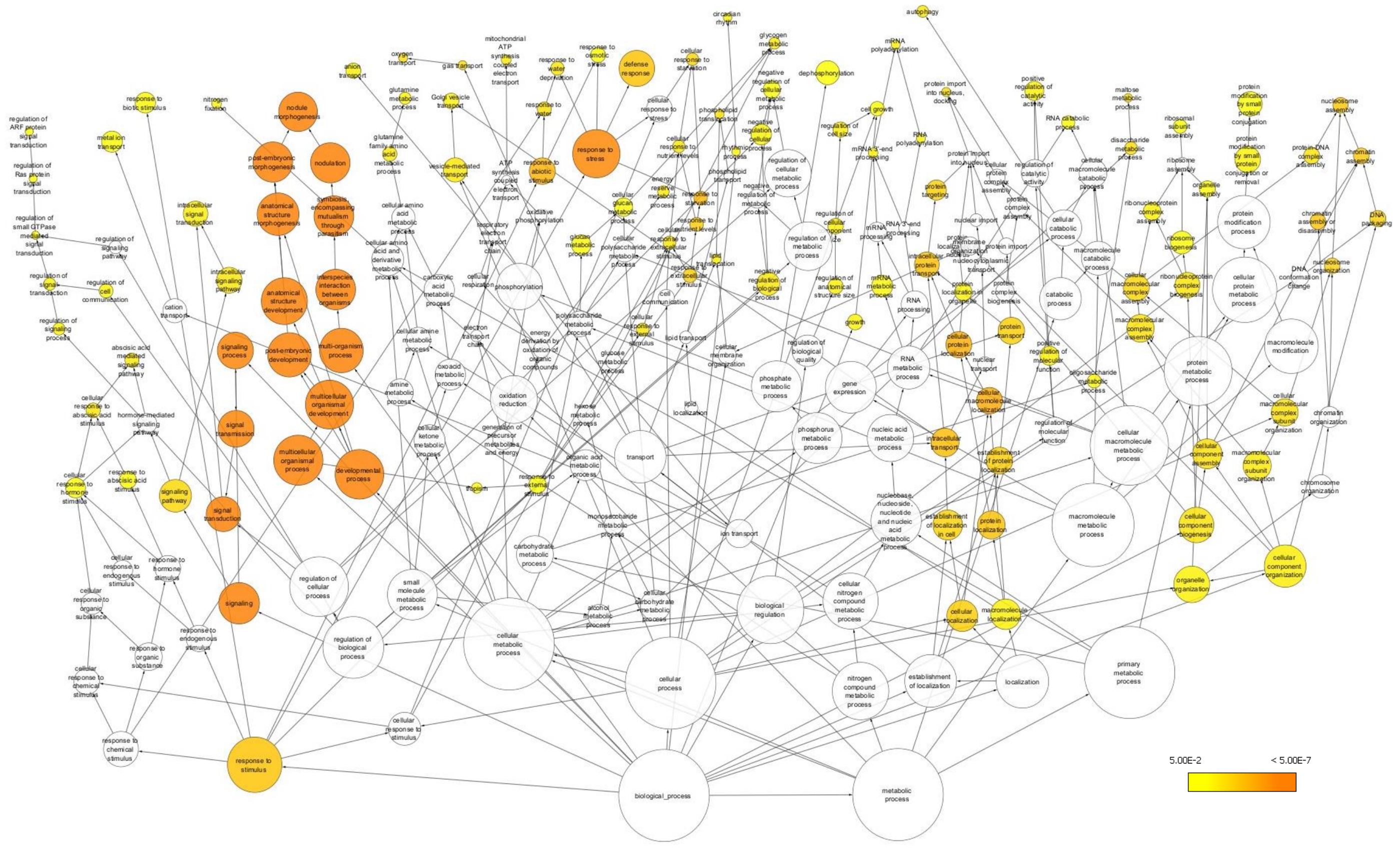

Supplementary Fig.S5. **Visualization of the over-represented Gene Ontology categories (GOs) in PEG_DATs using Cytoscape and BiNGO packages.** Colored nodes represent GOs that are over-represented based on a hypergeometric test and a Benjamini & Hochberg False Discovery Rate (FDR) correction with a p-value < 0.05 using the whole genome as reference (see the color legend p-value range bar below). Uncolored nodes are not over-represented, but are the parents of overrepresented categories.

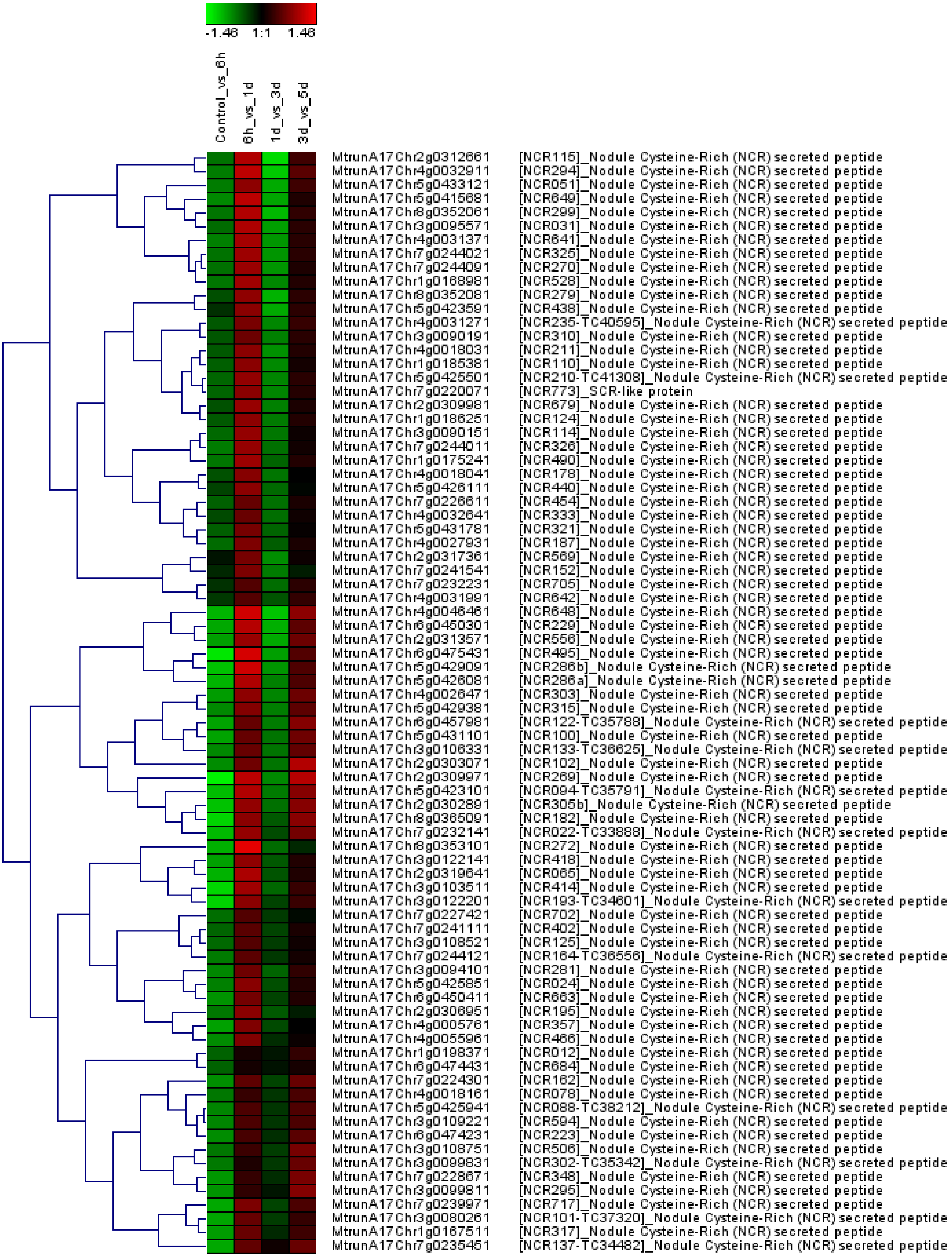

Supplementary Fig.S6. Heat map of transcript accumulation fold changes of N&PEG_DATs encoding Nodule Cysteine Rich (NCR) peptides. The hierarchical clustering was established using the Ward linkage method using the Genesis software.

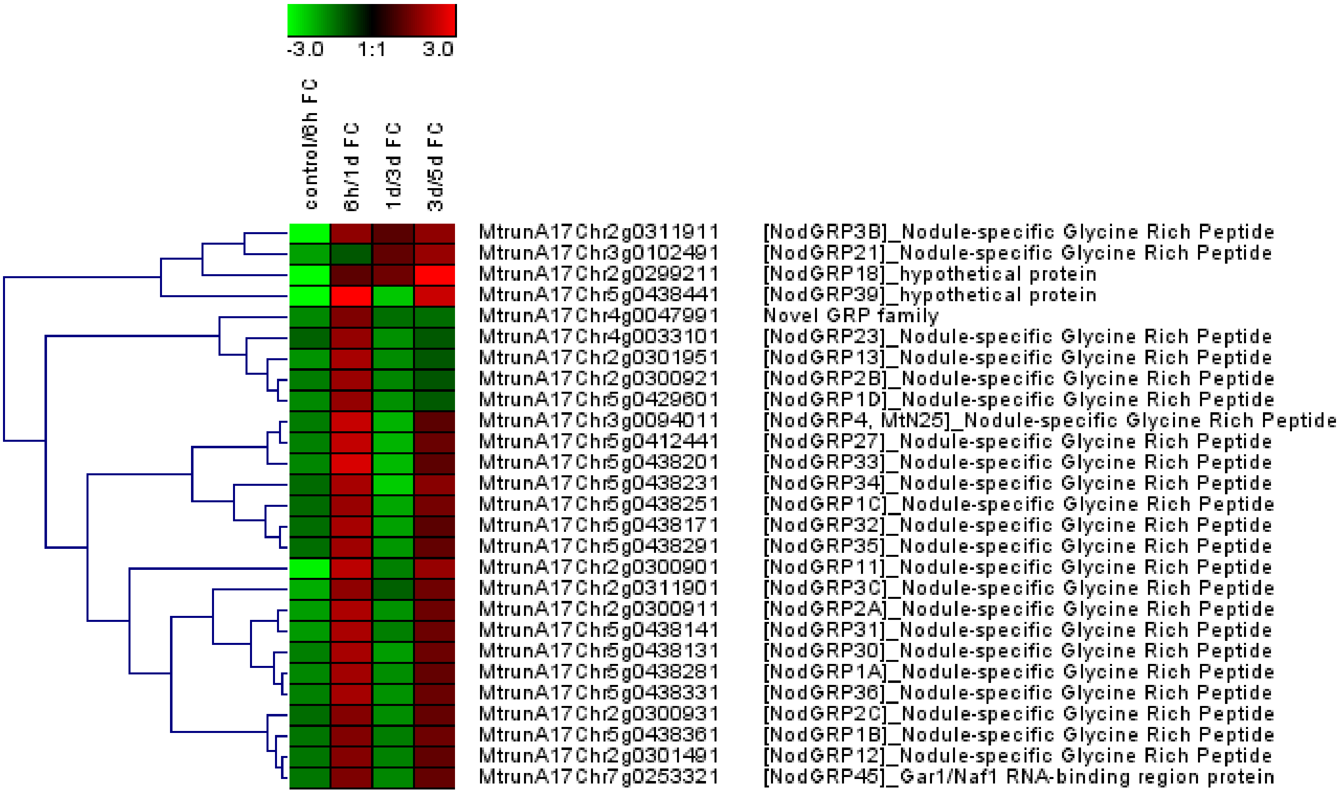

Supplementary Fig.S7. Heat map of transcript accumulation fold changes of N&PEG_DATs encoding Glycine Rich Peptides (GRPs). The hierarchical clustering was established using the Ward linkage method using the Genesis software.

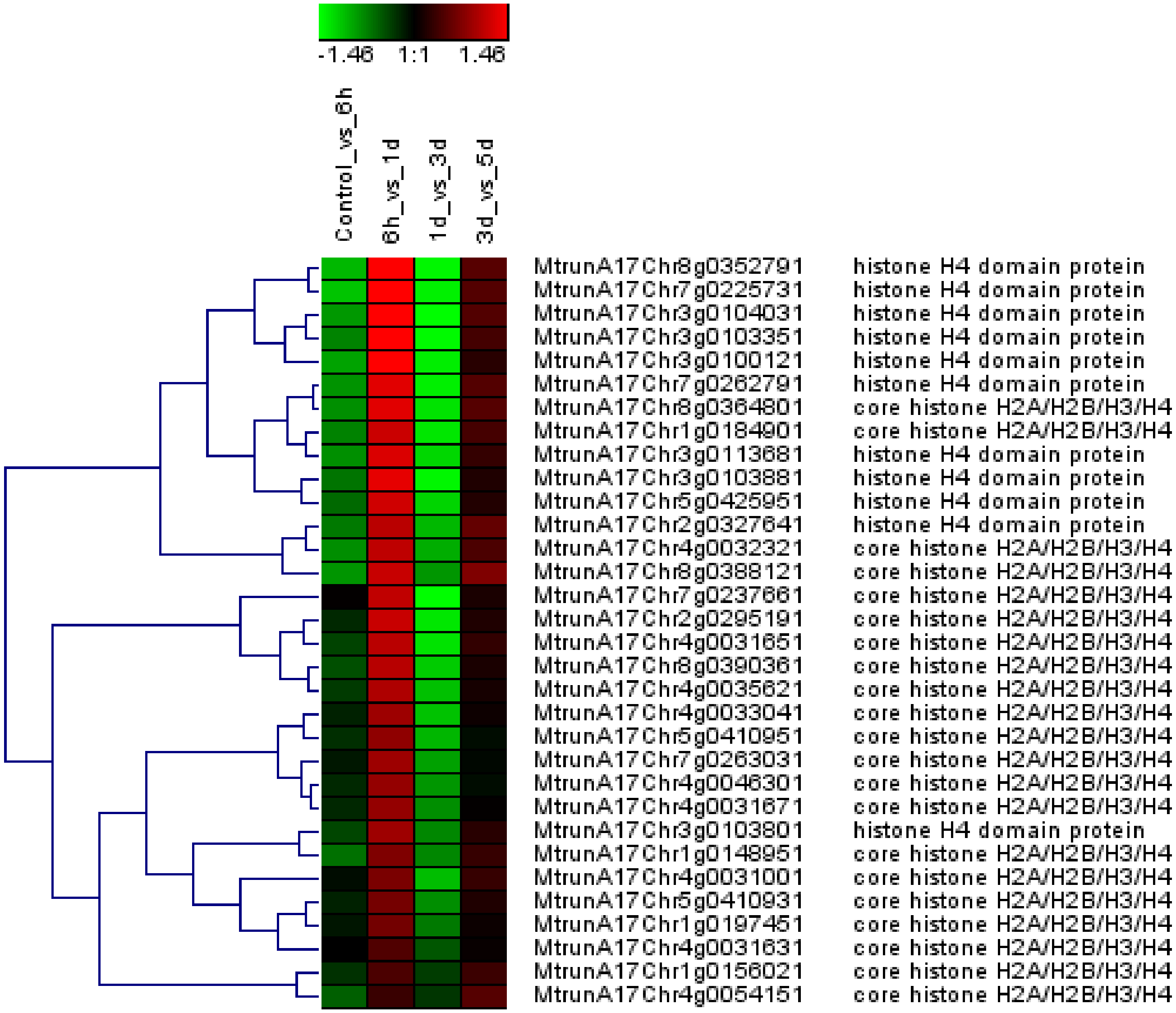

Supplementary Fig.S8. Heat map of transcript accumulation fold changes of N&PEG_DATs encoding core histone proteins. The hierarchical clustering was established using the Ward linkage method using the Genesis software.

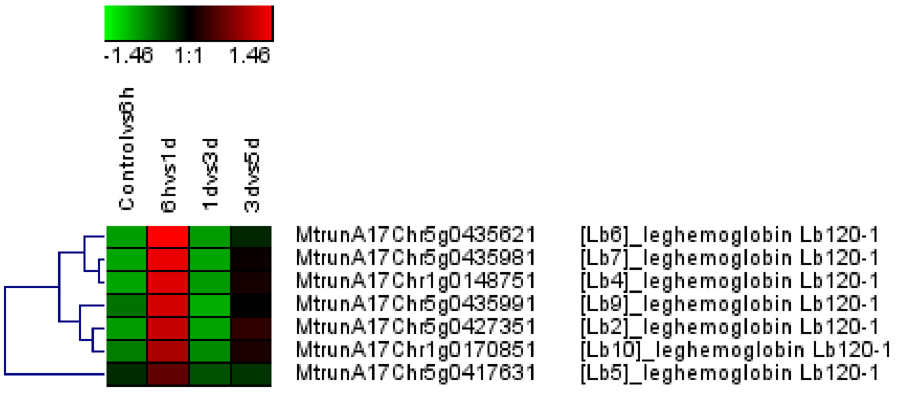

Supplementary Fig.S9. Heat map of transcript accumulation fold changes of N&PEG_DATs encoding Leghemoglobins. The hierarchical clustering was established using the Ward linkage method using the Genesis software.

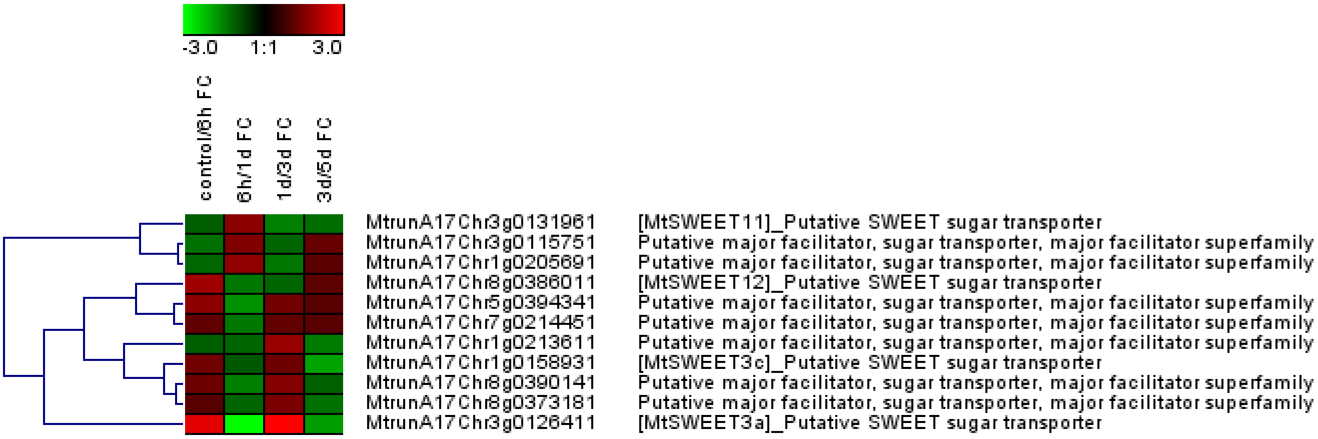

Supplementary Fig.S10. Heat map of transcript accumulation fold changes of N&PEG_DATs encoding sugar transporters. The hierarchical clustering was established using the Ward linkage method using the Genesis software.
